# The study protocol of a cluster-randomised controlled trial of family-mediated personalised activities for nursing home residents with dementia

**DOI:** 10.1186/1471-2318-12-2

**Published:** 2012-01-12

**Authors:** Eva S van der Ploeg, Cameron J Camp, Barbara Eppingstall, Susannah J Runci, Daniel W O'Connor

**Affiliations:** 1 Aged Mental Health Research Unit, Monash University, Melbourne Australia. Postal address: Kingston Centre, Warrigal Road, Cheltenham, Vic 3192, Australia

## Abstract

**Background:**

Following admission to a nursing home, the feelings of depression and burden that family carers may experience do not necessarily diminish. Additionally, they may experience feelings of guilt and grief for the loss of a previously close relationship. At the same time, individuals with dementia may develop symptoms of depression and agitation (BPSD) that may be related to changes in family relationships, social interaction and stimulation. Until now, interventions to alleviate carer stress and BPSD have treated carers and relatives separately rather than focusing on maintaining or enhancing their relationships. One-to-one structured activities have been shown to reduce BPSD and also improve the caring experience, but barriers such as a lack of resources impede the implementation of activities in aged care facilities. The current study will investigate the effect of individualised activities based on the Montessori methodology administered by family carers in residential care.

**Methods/Design:**

We will conduct a cluster-randomised trial to train family carers in conducting personalised one-to-one activities based on the Montessori methodology with their relatives. Montessori activities derive from the principles espoused by Maria Montessori and subsequent educational theorists to promote engagement in learning, namely task breakdown, guided repetition, progression in difficulty from simple to complex, and the careful matching of demands to levels of competence. Persons with dementia living in aged care facilities and frequently visiting family carers will be included in the study. Consented, willing participants will be randomly assigned by facility to a treatment condition using the Montessori approach or a control waiting list condition. We hypothesise that family carers conducting Montessori-based activities will experience improvements in quality of visits and overall relationship with the resident as well as higher self-rated mastery, fewer depressive symptoms, and a better quality of life than carers in the waiting list condition.

**Discussion:**

We hypothesise that training family carers to deliver personalised activities to their relatives in a residential setting will make visits more satisfying and may consequently improve the quality of life for carers and their relatives. These beneficial effects might also reduce nursing staff burden and thus impact positively on residential facilities.

**Trial Registration:**

Australian New Zealand Clinical Trials Registry - ACTRN12611000998943

## Background

Both people with dementia and their family carers may suffer from the consequences of dementia such as confusion, disorientation and reduced language fluency. As a result, people with dementia may experience increasing difficulty in communicating their needs to family and professional caregivers for physical comfort, social engagement and meaningful activity. According to the *unmet needs theory *[1], the resulting distress contributes to the behavioural and psychological symptoms of dementia (BPSD). The psychological symptoms include anxiety, depression, hallucinations and delusions. Behavioural symptoms include restlessness, aggression and calling out. BPSD reach a peak in the middle to late phases of dementia and greatly increase the likelihood of admission to an aged residential facility [2]. Within facilities, rates of BPSD are often high. In 11 Sydney nursing homes, for example, 60% of residents had an affective disorder, 53% showed activity disturbance and 77% were aggressive [3].

When BPSD stem from pain, co-morbid major depression or psychosis, treatment with analgesics, antidepressants or antipsychotics can bring relief. However, in cases where the causes of BPSD are less obvious, medications have variable efficacy and can lead to adverse effects such as worsened confusion and falls [4,5]. More attention is being paid, therefore, to non-pharmacological interventions that better address the needs that underpin BPSD. In two recent systematic reviews, it was demonstrated that treatments such as music, recreation therapy and relaxation therapy significantly reduced BPSD with effect sizes ranging from 0.5 to 1.15 [6,7]. The largest effects were found when treatments were tailored to individuals' own backgrounds, interests and skills.

Two studies conducted since the reviews suggested that Montessori-based activities significantly reduced agitated behaviours and increased positive mood and engagement [[8], van der Ploeg ES, Eppingstall B, Camp CJ, Runci S, Taffe J, O'Connor DW. The effect of personalised, one-to-one interaction using Montessori-based activities on agitation, engagement and mood in aged care facility residents with dementia. Submitted]. Montessori-based activities follow the principles espoused by Maria Montessori and subsequent educational theorists who promoted engagement in learning by sequencing tasks from simple to complex; providing cues to successful completion; encouraging repetition, and carefully matching demands to individuals' interests and levels of competence [9,10]. For people with dementia, Montessori-type programs entail detailed interviews with family carers about the resident's former interests and skills coupled with assessments of cognitive, language and motor skills. A range of activities are then presented, tested and refined. When dementia is advanced, the activities are simple (e.g. completing a jigsaw made from a family photograph). Facilitators present tasks deliberately, modelling them first and using little language if appropriate. The main objective is to engage participants' interest and involvement. Despite a growing evidence base, personalised one-to-one activities are rarely implemented in aged care facilities as they require extra time and resources that are often not available.

Dementia may also inflict negative consequences for family members of the individual with dementia [11]. Frequently reported adverse consequences of caring for relatives with dementia include physical health problems, depression, social isolation and financial burden [12]. These stresses might be expected to diminish following admission to a residential facility where care tasks are undertaken by paid staff. In reality, stress levels often remain high, with only a change in the source of burden [13,14]. New stressors may include the practicalities of visiting, tension with staff regarding care roles, concerns about care and continuing costs [15]. Carers possibly also experience guilt and difficulties relinquishing their role as primary supporter [16,17]. In addition, visits to the facility can prove unsatisfying, especially if the resident is anxious, agitated and increasingly more confused. There are fewer opportunities for easy communication, shared activities and contentment in the relationship [18,19]. Interventions to ease this transition have included carer education and support groups [20]. While promising, they considered the family carer and resident separately, overlooking the loss of relationship between the two, particularly significant for spouses [15,21]. Preserving and enhancing this relationship through meaningful activities can restore a sense of competence and self-determination for family carers [22] and improve the well-being of both parties [23]. An activity program delivered by family carers to relatives with dementia living in the community resulted in large increases in carers' confidence through perceived enhanced skills and personal control as well as enhanced engagement and mood of the individuals with dementia [24].

Interventions to alleviate carer stress and BPSD have mostly treated carers and relatives separately rather than focusing on re-establishing and enhancing their relationship. Since one-to-one structured activities have been shown to reduce BPSD and also improve the caring experience, they seem an ideal intervention to bring people together and thus improve wellbeing for both carers and their relatives with dementia. The current study will investigate the effect of individualised activities based on the Montessori methodology administered by family carers in residential care on the wellbeing of carers. We hypothesise that family carers conducting Montessori-based activities will experience higher self-rated mastery, fewer depressive symptoms, and a better quality of life than carers in a waiting list period, as well as a better quality of visits and overall relationship with the resident.

## Methods/Design

### Study design

We will employ a cluster-randomised trial to allocate a condition (treatment vs. waiting list) at facility level. The project protocol has been approved by the ethical committees of Monash University and Southern Health.

### Study protocol

The study period will be brief to reduce the likelihood that changes in outcomes are due to dementia progression, inter-current illness or other incidental events. Activity sessions must be long enough to detect changes in selected outcomes during the carer's visit, but not so long that sessions are likely to be interrupted by meals or nursing interventions. In our recently completed Montessori study [25], the choice of 30-minute sessions has worked well. A study period of up to four weeks has also proved feasible. We will therefore employ an exposure of six sessions spread over a three week period for both conditions. The choice of condition (treatment or control) will be randomly determined using Excel. The program from week 1 (W1) to week 5 (W5) is shown in Table 1. Baseline measures will be conducted at the start of the Montessori workshop or dementia education session (W1). All participants will receive a follow-up phone call half way through W2-W4 to ascertain they have filled out questionnaires after each visit. A face-to-face appointment will be made to complete the final measurements at the end of W4.

**Table 1:** Program for treatment and control condition groups

	Treatment group	Control group
W1	Group Montessori workshop	Dementia education session

	↓	↓

W2 W3 W4	6 Montessori sessions	Waiting list period

		↓

W5		Group Montessori workshop

### Ethical considerations

A facility staff member will identify potential residents and their carers and seek agreement from the carer to be contacted by the research team. Since the residents concerned will have dementia, often to a moderate or severe degree, the family carer (or significant other person) who visits most frequently will be asked for written informed *consent*, on his/her own behalf and on behalf of the person with dementia, in accordance with both the Monash University HREC and the Victorian Civil and Administrative Tribunal. Persons with dementia must also *assent *to participate in the study. If it is clear from their demeanour or behaviour that they do not wish to participate, their participation in the study will be discontinued.

Carers will either directly commence with the Montessori training and implementation or will be told that due to limited resources they will be put on a waiting list for four weeks (the control condition). We will ask them to complete the baseline and all follow-up measures and to attend a dementia education session. Carers will be blinded to the hypotheses of the study and the purpose of the waiting list option. After the carers in this condition complete the measurements associated with the control period, they will be debriefed regarding the purpose of the waiting list option and are invited to participate in a Montessori workshop.

### Setting

The study will be conducted in mainstream and psychogeriatric aged care facilities in the state of Victoria, Australia. Preference will be given to larger facilities (≥ 60 beds).

### Recruitment

Aged care facilities will be contacted by telephone to explain the studies. If facilities show interest in the study, researchers will visit the facility to provide all relevant parties (director of nursing, nursing staff and diversional therapists) with more detailed information. If facilities consent to participate in the study, a pre-selection screening will be conducted to identify eligible participants. An appointed delegate of the facility will then contact the identified family carers to ask if they consent with forwarding their contact details to a Monash University researcher. When verbal consent is given a senior researcher will contact the carer to explain the study and answer queries. If carers express interest in the studies they will be sent a Participant Information and Consent Form package. Once written consent is received, researchers will gather baseline information and plan the training session with groups of up to six participants. If more than six family carers are eligible and interested in the study in a particular facility, more training sessions will be organised.

### Participants

#### Inclusion criteria for residents

(i) A chart diagnosis of dementia or probable dementia; and (ii) residency in the facility for ≥ 3 months to allow for adjustment to the new setting.

#### Exclusion criterion for residents

Acute life-threatening illness as reported by nursing staff.

#### Inclusion criteria for family carers

(i) Visit regularly ≥ 2 times per week for 30 minutes or more to match study requirements, (ii) willing to follow study protocol, and (iii) sufficient fluency in English to understand the workshop and fill out questionnaires.

### Experimental conditions

#### Montessori-based activities

Family carers will be trained in the principles underpinning Montessori activities in three-hour group sessions in their relative's facility. A pair of researchers will conduct these workshops. They will spend half an hour helping carers to fill out the baseline questionnaires; an hour explaining the theoretical basis of Montessori activities in dementia, and an hour and a half in smaller groups brainstorming and practising possible activities.

Carers will choose up to 10 activities based on their relative's former interests and current motor and language skills. For the purpose of continuity of the intervention after the study finishes, carers will be asked to prepare materials using their own and the facilities materials. The mode of delivering activities can be easily adapted to residents' cognitive status, making it possible to include people with mild through to very severe dementia. For example, a suitable activity for a former mechanic with moderate dementia might include discussing the characteristics and qualities of period cars shown in laminated photographs. If dementia is severe, the activity can be simplified to sorting the photographs into categories based on colour, or preference.

#### Control condition

The control condition should match the level of contact with the researchers and additionally control for the effects of the carers meeting each other as part of the group training. To ensure comparability with the experimental condition, the family carers will receive a dementia education session lasting three hours. Again, the first half hour will be spent filling out the baseline questionnaires; an hour and a half will be used for dementia education after which the groups will split into smaller groups to discuss the material just presented. To match the involvement of the relatives with dementia, we will ask carers towards the end of the training session to spend some time with their relative. During this time, the researchers will spend time with every couple, introducing themselves to the residents and engaging in general conversation with them.

### Outcomes measures

#### Primary measure

##### Quality of visits

We will ask carers to rate their overall satisfaction with each study visit on a 5-point Likert scale. In addition, they will complete a truncated version of the Pearlin Mastery Scale [26] adapted to care-related situations [27], focussing on their sense of personal mastery during each visit. Pioli (2010) [27] deleted two items of the original scale, because they were not specifically related to carer interactions: 'Sometimes I feel that I'm being pushed around in life' and 'What happens to me in the future mostly depends on me'. The remaining items will be reworded to make them relevant to visits, for example 'I had little control over the things that happened today when I spent time with my family member' and 'I felt helpless in dealing with the problems I experienced during my visit today'.

#### Secondary measures

All of the following secondary measures will be completed at baseline and at the end of the three-week study period.

##### Carer-resident's quality of relationship

Family carers will be asked to rate the overall quality of their relationship with their relative on a five-point Likert scale. They will also complete the 15-item Mutuality Scale of the Family Caregiving Inventory which measures the positive aspects of relationship quality using the dimensions love, shared pleasurable activities, shared values, and reciprocity [28]. Examples of items are "How close do you feel to your relative?" and "To what extent do you enjoy the time the two of you spend together?" The scale has high internal consistency (*alpha *0.91-0.95) in studies of family care [29] and scores correlate highly with carer role strain [28] and the stages of Parkinson's disease [29].

##### Carer's mastery

Carers will complete five items of the Pearlin Mastery Scale matching the items administered at the end of every study visit but using the scale's original wording describing global mastery [26]. These items include, "I have little control over the things that happen to me" and "There is really no way I can solve some of the problems I have". This shortened scale has a high internal consistency with an *alpha *of 0.72 [27].

##### Carer's mood

The widely used Center for Epidemiological Studies Depression Scale (CES-D) is a 20-item self-report scale of depressive symptoms covering mood, self-esteem, energy, relationships, sleep and appetite [30]. A cut-off point of ≥ 16 is often used to denote clinically relevant depression. Internal consistency is high with an *alpha *of 0.85-0.92 and scores correlate well with clinicians' ratings and other measures of depression, distress and negative affect.

##### Carer's quality of life

The Carer-QoL [31] is a short, seven-item self-report questionnaire concerning difficulties with physical and mental health, finances and social support. Scores correlate highly with measures of carer strain, carer burden and other global measures of their quality of life including the EuroQol-5D. Feasibility has been reported as excellent with 98% of subjects completing all items.

##### Other measures

Measures collected at baseline will include residents' and carers' socio-demographic details, type of relationship (friends and neighbours can be included if they meet criteria), duration of caring, the frequency and length of visits, and quality of the pre-morbid relationship. We will abstract the date of admission to the facility from the resident's file and will complete the Clinical Dementia Rating Scale (CDR) [32] and Cohen Mansfield Agitation Inventory [33] with a staff member to establish dementia severity and level of agitation.

### Sample size calculation

The sample size calculation is based on a study comparing wives caring for moderately impaired husbands that exhibit probable Alzheimer's disease with wives whose spouses demonstrate no mental or physical condition matched on sociodemographic background [34]. We calculated means per group (carers vs. non-carers) for the total Mutuality Scale of the Family Caregiving Inventory [28]. Caregiving wives reported a score of 2.89 and the control participants a score of 3.48 (pooled SD = 0.74), with higher scores indicating a higher quality of the relationship (effect size 0.80). We have used G*Power 3.1.2 (test family = t-test, statistical test = means: difference between two independent means (two groups), alpha = 0.05, power = 0.80 and effect size *d *= 0.80) to calculate that 21 participants in each condition would suffice to reveal such a difference. We estimate 20% attrition and will recruit 50 carers.

### Statistical analysis

We will use independent t-tests to compare the means of outcome measures for the Montessori and control conditions. Next, each outcome measure will be subjected to a 2-way ANOVA with condition (Montessori *vs*. control) as fixed factor. We will then undertake ANCOVAs to investigate whether the intervention enhances certain outcomes (e.g. quality of relationships) after controlling for others (e.g. quality of pre-morbid relationships) to explore the possibility of mediation. Secondly, we will extend these models to examine whether other factors, such as resident-carer relationship (e.g. spouse *vs*. child) and dementia severity (moderate *vs*. severe) serve to moderate treatment benefits. Finally, we will conduct a multilevel variant of these ANOVAs to include higher level factors such as the aged care facility.

## Discussion

Montessori activities and other one-to-one interactions have successfully reduced agitation and increased positive affect and engagement in people with dementia. Helping family carers to deliver these activities in a residential setting is likely to make visits more satisfying and improve the quality of life for both the carers and the care-recipients. These beneficial effects might also reduce nursing staff burden and thus impact positively on residential facilities.

## Competing interests

The authors declare that they have no competing interests.

## Authors' contributions

EvdP conceived the original research idea and developed the exact study procedure with input from her co-authors. CC has adapted the Montessori methodology for people with dementia and commented on the Montessori component of the study. BE, SR and DOC have extensive experience with conducting studies in aged care settings and assisted with methodological decisions such as study sample and choice of outcome measures. EvdP has written this article. All authors commented on a draft and read and approved the final version of the paper.

## Pre-publication history

The pre-publication history for this paper can be accessed here:

http://www.biomedcentral.com/1471-2318/12/2/prepub
